# Fulminant Rhabdomyolysis and Acute Kidney Injury Associated With Pontiac Fever: A Case Report

**DOI:** 10.7759/cureus.103332

**Published:** 2026-02-10

**Authors:** Roman Khomynets, Yolanda S Pereira, David Pires, Cristina Alcântara

**Affiliations:** 1 Department of Internal Medicine, Local Health Unit of Santa Maria, Lisbon, PRT

**Keywords:** acute kidney injury, creatine kinase, legionella pneumophila, non-pneumonic legionellosis, non-traumatic rhabdomyolysis, pontiac fever, rhabdomyolysis causing acute kidney injury

## Abstract

*Legionella pneumophila* typically manifests in two distinct clinical forms: Legionnaires' disease, characterized by severe pneumonia, and Pontiac fever (PF), a non-pneumonic, influenza-like illness that is usually self-limiting within 2-5 days. While PF is characterized by a high attack rate among exposed individuals in outbreak settings, its true incidence remains unknown and is likely underdiagnosed. Consequently, in routine surveillance data, Legionnaires' disease accounts for the vast majority of reported *Legionella* infections, with PF representing only a negligible fraction. We report a rare case of a 24-year-old man who presented with high fever, myalgia, and gastrointestinal distress, subsequently developing fulminant rhabdomyolysis. Laboratory findings were remarkable for a peak creatine kinase (CK) level of 737,860 U/L and Kidney Disease: Improving Global Outcomes (KDIGO) stage III acute kidney injury (AKI) requiring renal replacement therapy. Despite the complete absence of pulmonary involvement or hypoxemia, serological testing performed during outpatient follow-up retrospectively confirmed *Legionella* infection. This case emphasizes that PF, despite its reputation as a mild illness, can rarely precipitate life-threatening systemic muscle destruction and multi-organ dysfunction.

## Introduction

*Legionella pneumophila* is an aerobic, Gram-negative bacillus with 52 species and 70 serotypes. It is the causative agent of two primary clinical syndromes: Legionnaires' disease, characterized by acute pneumonia, and Pontiac fever (PF), a non-pneumonic, influenza-like illness [1-3]. Epidemiologically, these forms differ significantly: while PF is associated with high attack rates in outbreak settings (often exceeding 90%), Legionnaires' disease accounts for the vast majority of notified cases in routine surveillance, likely due to the underdiagnosis of the milder form. Rhabdomyolysis, characterized by the necrosis of skeletal muscle resulting in the release of intracellular contents such as creatine kinase (CK) and myoglobin into the circulation, is a recognized complication of *Legionella *infection. However, such massive myocytic destruction is predominantly associated with the pneumonic form of the infection [4]. The occurrence of catastrophic rhabdomyolysis in the absence of pneumonia (Pontiac fever) is exceedingly rare and poses a significant diagnostic challenge for clinicians.

## Case presentation

A 24-year-old man of Cape Verdean origin, residing in Portugal for four months, presented to the emergency department with a one-week history of high-grade fever (39.3°C), headache, generalized myalgia, arthralgia, abdominal pain, and watery diarrhea. The patient had no significant past medical history and was not taking any prescription or over-the-counter medications. He explicitly denied recent strenuous physical exertion, trauma, alcohol abuse, or illicit drug use. Furthermore, a urinary toxicology screen performed upon admission was negative, and there was no clinical evidence of seizures.

Physical examination was remarkable for generalized muscular edema (myoedema) and chromaturia. Vital parameters were within physiological limits, and the patient was normoxemic on room air.

Arterial blood gas analysis confirmed the absence of hypoxemia, showing a compensated non-anion gap metabolic acidosis.

Laboratory evaluation revealed massive rhabdomyolysis and severe acute kidney injury (AKI). The initial creatine kinase (CK) level was reported as >500,000 U/L, a value corresponding to the upper limit of quantification of the emergency department laboratory. Urinalysis was remarkable for leukocyturia and positive nitrites, alongside proteinuria and hematuria. Screening for *Legionella* urinary antigen and other common infectious etiologies was initially negative. The admission laboratory panel and its subsequent evolution are detailed in Table 1.

**Table 1:** Temporal evolution of laboratory parameters during hospitalization leading to renal replacement therapy ^1^Day of dialysis induction ^2^Value corresponding to the upper detection limit of the emergency laboratory assay

Laboratory parameters	Admission	Day 4	Day 6^1^	Reference value
Creatine kinase (U/L)	>500,000^2^	737,860	235,060	20-200
Creatinine (mg/dL)	1.99	6.36	7.71	0.7-1.2
Myoglobin (ng/mL)	19,266 ng/mL	-	-	<70
Aspartate aminotransferase (U/L)	2,998	2,990	-	8-33
Alanine aminotransferase (U/L)	625	823	-	19-25
C-reactive protein (mg/dL)	28.9	22.11	8.55	<0.8
Potassium (mmol/L)	5.9	5.7	5.7	3.5-5.1

**Figure 1:**
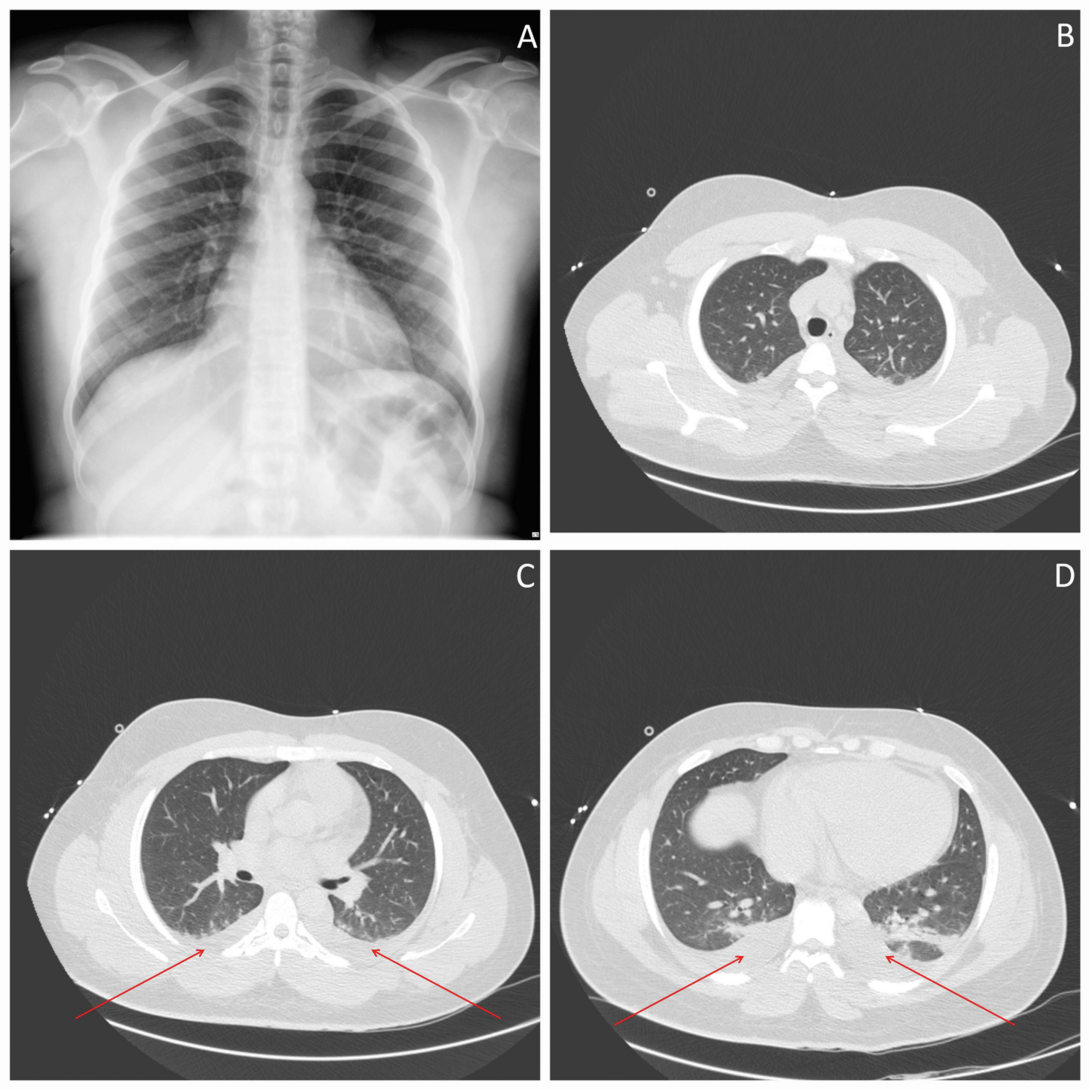
Thoracic imaging findings (A) Posteroanterior chest X-ray demonstrating clear lung fields with no obvious consolidations or infiltrates. (B-D) Sequential axial slices of chest CT from the upper to lower thorax. The scans confirm the complete absence of parenchymal pneumonia, infiltrates, or abscesses. Panel D reveals trace bilateral pleural effusions (posterior dependent densities) and generalized diffuse subcutaneous edema, consistent with the systemic presentation of Pontiac fever. CT: computed tomography

Imaging was crucial to exclude pulmonary involvement. A chest X-ray and computed tomography (CT) scan of the chest, abdomen, and pelvis showed no evidence of pneumonia, infiltrates, or consolidations, identifying only trace bilateral pleural effusions and diffuse subcutaneous edema (Figure 1).

The patient was diagnosed with fulminant rhabdomyolysis of indeterminate etiology and Kidney Disease: Improving Global Outcomes (KDIGO) stage III acute kidney injury (AKI). Therapeutic management prioritized aggressive isotonic fluid resuscitation alongside urinary alkalinization with 8.4% sodium bicarbonate, primarily targeting the correction of the documented non-anion gap metabolic acidosis and prevention of myoglobinuric renal tubular damage.

Concurrently, empiric antimicrobial coverage with intravenous ceftriaxone and doxycycline was initiated. Ceftriaxone was chosen based on admission urinalysis findings (leukocyturia and positive nitrites), while doxycycline was included to cover potential systemic infectious etiologies associated with rhabdomyolysis, including *Leptospira *spp. and *Rickettsia* spp.

However, despite falling CK levels after day 4, renal function initially deteriorated (Table 1), requiring five sessions of intermittent renal replacement therapy. Subsequently, the patient demonstrated a favorable clinical evolution.

An extensive workup was performed during hospitalization to exclude other etiologies. Serological and molecular testing yielded negative results for viral panels (including influenza A and B, human immunodeficiency virus, hepatitis B and C, cytomegalovirus, Epstein-Barr virus, parvovirus B19, adenovirus, and coxsackie/enterovirus), bacterial zoonoses (*Leptospira *spp., *Rickettsia *spp., *Coxiella burnetii*, and *Mycoplasma pneumoniae*), and parasitic infections (toxoplasmosis and malaria). Furthermore, autoimmune screening was negative, and electromyography combined with a muscle biopsy showed no evidence of immune-mediated or hereditary myopathies.

Notably, *Legionella *screening during the acute phase was limited to the urinary antigen test, which was negative. Consequently, the patient was discharged on day 15 with the etiology of rhabdomyolysis classified as indeterminate. At discharge, renal function had significantly recovered (creatinine: 1.92 mg/dL), and CK levels had normalized (218 U/L).

Diagnostic investigation continued in the outpatient setting to rule out atypical infections not detected by standard screening. At the first follow-up visit (three months post-discharge), serology revealed positive anti-*Legionella *antibodies via chemiluminescence immunoassay (IgG index: 2.25, IgM index: 1.14).

To confirm the diagnosis and assess antibody kinetics, serology was repeated two months later (five months post-discharge). The results demonstrated a progressive rise in IgG (index: 2.79) with persistent IgM positivity (index: 1.11). This evolution of antibody titers, specifically the rising IgG and persistent IgM in the convalescent phase, retrospectively confirmed the diagnosis of Pontiac fever.

## Discussion

This case highlights an exceptional clinical association between Pontiac fever and fulminant rhabdomyolysis. The most remarkable finding was the profound elevation of creatine kinase (peak level: 737,860 U/L) in a patient devoid of clinical or radiological signs of pneumonia, a presentation that effectively excludes Legionnaires' disease.

Diagnostic confirmation of Pontiac fever is often challenging. The urinary antigen test primarily detects *Legionella pneumophila* serogroup 1 and may yield false-negative results in infections caused by other serogroups or species [5,6]. Consequently, serological testing with demonstration of seroconversion remains an important diagnostic tool in selected cases [6,7].

The pathophysiology of muscle injury in *Legionella *infection remains incompletely elucidated. Proposed mechanisms include direct bacterial invasion of skeletal muscle, endotoxin-mediated toxicity, and cytokine-driven inflammatory damage [4,8,9]. The existence of *Legionella*-associated myositis has been documented, lending biological plausibility to severe extrapulmonary muscle involvement even in the absence of pneumonia [8].

Published reports of *Legionella*-associated rhabdomyolysis almost invariably involve cases complicated by established pneumonia [10-12], underscoring the exceptional nature of this non-pneumonic presentation. This case, therefore, challenges the traditional perception of Pontiac fever as an invariably mild and self-limited illness and demonstrates that it can, in rare circumstances, precipitate life-threatening systemic complications.

## Conclusions

Pontiac fever should be considered in the differential diagnosis of severe unexplained rhabdomyolysis, even in the absence of respiratory symptoms or hypoxemia. While typically a mild and self-limiting illness, this case demonstrates that it can, in rare instances, precipitate life-threatening systemic complications. Early recognition, careful diagnostic evaluation, and prompt supportive management, including aggressive fluid resuscitation and timely renal replacement therapy when indicated, are essential to optimize outcomes in such atypical presentations.
